# Controlling strain localization in thin films with nanoindenter tip sharpness

**DOI:** 10.1038/s41598-024-77457-9

**Published:** 2024-10-26

**Authors:** Stanislav Zak

**Affiliations:** Erich Schmid Institute of Materials Science, Austrian Academy of Sciences, 8700 Leoben, Austria

**Keywords:** Finite element modelling, Nanoindentation, Strain localization, Thin film, Thin multilayer, Structural properties, Mechanical engineering, Characterization and analytical techniques

## Abstract

Thin film nanoindentation has increased interest due to its usage in various applications. It is virtually impossible to measure thin film elastic modulus without the substrate influence. Several different methods exist to obtain the true thin film’s elastic modulus with no attention given to investigate what parameters can improve insight into thin film mechanical property measurement. A key parameter is the tip radius. This work is aimed at quantifying the influence of the tip radius on the strain field under the indenter. Three Berkovich indentation tips with different tip radii were used for thin multilayer nanoindentation with numerical modelling. The results confirm the existence of the large elastically deformed zone, with a strong localization under the tip. Comparison between the experiments and numerical model shows direct connection between the tip radius and strain localization affecting the experiment, emphasizing importance of knowing the tip radius.

## Introduction

In the world of modern material science, the development, use and investigation of thin films as functional or protective part of technological applications is a hot topic (see e.g. recent review articles^1–5^). However, to properly work with modern thin films and coatings, it is crucial to know their mechanical properties, allowing the assessment of their durability, deformation under load, fracture properties, lifetime, compatibility with other materials (as substrates or multilayers). Throughout the years, several methods to measure thin films’ elastic and plastic mechanical properties were devised^6–8^. A standard mechanical testing such as tensile and compression tests, or beam bending can be adopted into the micro-scale throughout the pillar compression testing^9,10^, micro-tensile tests^11–14^, beam (cantilever) bending tests^12,15^. Additionally, thin films’ general bending compliance allows to use specialized bulge testing procedure using a 3D deformation under uniform pressure^16–18^. In special cases, more complex and sophisticated methods can be used, such as contact resonance with use of atomic force microscopy (AFM)^19,20^ or micromechanical spectroscopy performed on cantilevers cut from the thin film^21^. These methods are inherently slow to use (do not support high-throughput testing) and complex in sample preparation involving extensive focused ion beam (FIB) or similar milling for compression and bending testing and the time and resource intensive preparation of testing windows for bulge testing. Micro-tensile tests are sensitive to sample geometry and thickness and AFM techniques provide a lot of instabilities in calibration and results analysis. In addition to these methods, nanoindentation has been extensively used for 30 years. Nanoindentation consisting of indenting the material with a sharp indenter, recording the whole load-displacement curve and using nano- to micro-scale displacement^22^ allows for a straightforward and high-throughput-capable method to measure the hardness and Young’s modulus of the tested material. The small scale of such testing directly calls for use on thin films and coatings^23–25^. However, such nanoindentation cannot be performed on free-standing films and therefore, it is strongly influenced by the substrate effect.

Besides standard, well known OliverPharr method^22^ (which needs a precise tip calibration prior the indentation measurements) with single indents, there are additional methods for use with nanoindentation. One is JoslinOliver approach^26^, which does not need a tip area calibration, however, the indented material hardness must be known “a priori”. The other approach would be continuous stiffness measurement (CSM)^27^, which probes the material mechanical properties as a function of indentation depth in one location. In comparison to OliverPharr method, the CSM cannot be considered quasi-static. Due to this reason, and fact that the JoslinOliver approach is based on previous knowledge of hardness and is not widely used, the presented results in this manuscript will be obtained via OliverPharr method. It should be noted that nanoindentation scratching is additional valid test of material properties and can be adapted to micro-scale^28,29^. However, it is mostly used to obtain fracture properties of thin films or the adhesion energy and delamination crack propagation^29^. From the basic mechanical properties of the thin films, it can be used only to evaluate contact hardness^28^, not the general elastic and plastic material properties of the film itself. Therefore, it will not be considered in the presented work.

The early works in thin film nanoindentation field adopted the 50-year-old 10% rule by Bückle^30^, stating that indentation to the depths of less than 10% of the thin film thickness should be free of the substrate effect. However, the original work by Bückle dealt only with the hardness and for materials on fairly stiff substrates. Therefore, it cannot be used for elastic modulus measurements, especially in conjunction with soft substrates, which is supported by findings of Xu and Rowcliffe^31^ (“…there is no universal critical penetration depth for measuring the mechanical properties of films. The critical penetration depth depends on the combination of the mechanical properties of the film/substrate system.”) or Bull^32^, finding that for elastic modulus measurements, the indentation depths less than 1% of the film thickness should be considered. In last decades, some works pushed the nanoindentation of thin films forward by improving the nanoindentation results analysis for pure film hardness^33–35^ and elastic modulus^32,34–41^. For example, work by Saha and Nix^35,36^ employed method by Joslin and Oliver (J–O)^26^ where with known (or previously measured) hardness, *H*, the elastic modulus, *E*, of the film in question can be calculated. This model works because it also removes pile-up effects, however, it assumes the same microstructure of the thin film for comparable results. But this is not always the case for the different substrates. Due to surface energy differences, a constant microstructure should not always be assumed for any film-substrate combination, thus the hardness measured may not be the same for the different substrates. Ding et al.^40^ employed numerical modelling to describe influence of elastic moduli ratio between the film and substrate on the load during the nanoindentation and stresses, however, the resulting explicit formulas relates only to extremely soft films from biological materials. Saha et al.^37^ looks into hardening effects for soft film on hard substrate system. Their results show again the dependence of the measured Young’s modulus and hardness on the indentation depth despite the soft-on-hard material system. A rather complex model was also introduced by Li and Vlassak^39^ with use of the solution by Yu for the elastic contact of an indenter. This model can be applied to ultra-thin films over a large indentation depth, but it is precise enough only with use of large number of indents or using the dynamic indentation. Zhao et al.^41^ used numerically calculated weight functions to compensate for the substrate effects, clearly showing the transition between the thin film and substrate elastic moduli. However, their approach needed extensive modelling for each individual case. All these models are rather complex for use in every lab/institute and require rather specific technique usable only on some material systems. Therefore, the most straightforward method to approximate the thin film material properties via nanoindentation is to perform large number of indents into both thin film and also the substrate to uncover the transition of the measured values between pure substrate and presumably the thin film. This transition can be than used for extrapolation to the zero-contact depth, which should theoretically match with the thin film real property, such as Young’s modulus^42^.

Our initial work on nanoindentation of the Mo and MoTa thin films on Si substrate^43^ uncovered the direct connection between the elastic and plastic deformation field size and the measured Young’s modulus and hardness, respectively, via the numerical modelling. The measured results went along with the previously mentioned works and showed undisputable difference between the elastic modulus and hardness measurements in terms of the depth where the actual influence of the indenter can reach, showing the real extent of the short-range plastic zone related to hardness and long-range elastic zone related to Young’s modulus. While the results also proved the necessity for very shallow indents (less than 2.5% of the film thickness) for elastic modulus measurement, the extrapolation of the results towards zero contact depth was not as straightforward due to large scatter of the results and not clear shape of the extrapolation function. This problem could be solved by a “brute force” approach with an increased number of used indents for shallow indentation depths. However, such a process would not be recommended due to the general need for speeding-up of the experiments and the risk of invalidating results by wrong calibration function due to the tip blunting^44^, whereas the intermittent tip calibration would furthermore increase the experimental time and render the test procedure impractical. The next exploitable finding from the mentioned research was that the transition of the results always follows the direction from the substrate value and is dependent on the ratio between elastic and plastic properties of the thin film and substrate. Therefore, use of the same thin films on different substrates could provide more quantifiable data with a smaller number of indents. This was demonstrated in^45^ where thin coatings of nano-Cu and CuZr metallic glass as well as their multilayered combinations were indented on glass and Si substrates. The nanoindentation showed that the extrapolation function for measuring the elastic modulus for zero contact depth can be a simple linear function and with the use of different slopes (with glass and Si substrates being on the opposite sides of the expected values for the coating) and different extent (with the multilayer coatings engineered to have different hardnesses) a more precise evaluation of the coating’s elastic modulus could be made.

While the extrapolation method with use of different substrates for the same thin film leads to precise results, it still needs an extensive indentation session taking off the time and life of the nanoindenter tip by blunting it. Therefore, it still needs more refinement to be able to use it on a practical daily basis. This can be done via tailoring the nanoindentation parameters to enlarge or shrink the elastic deformation field size. For it to be used for a large variety of samples, it should be achieved only by changing the parameters on the nanoindenter side. This leaves the option open with the indentation speed and the tip geometry. While the indentation speed has a large effect on nanoindentation (especially of the rate-dependent materials), it is a complex parameter of nanoindentation where also the indented material plays a role as well. Also, it is a well-known phenomenon, researched from a large number of different angles (see e.g^46–48^). Therefore, it is not a proper parameter to be investigated on a basic level for devising a better method for thin film nanoindentation in general. The tip geometry, on the other hand, offers more opportunities to uncover hidden characteristics of the indent. Currently it is widely assumed that with a proper tip contact area calibration, the nanoindentation with any tip shape is equal. This is mostly true if the size-scale of the deformed region under the tip is not a parameter influencing the results, such as bulk nanoindentation. The problem lies in the shape and extent of the deformed region – it is strongly dependent on the tip shape. Qiao et al.^49^ shows that the tip bluntness directly influences the indentation size effect for hardness measurements through the different number of geometrically necessary dislocations. Research by Ochoa and Breitkopf^50^ shows a strong influence of the diameter of the flat indenter via numerical modelling and the work by Sen and Sujith^51^ points out the differences between the sharp and spherical tips as well as the influence of the spherical tip radius. These lead to the conclusions that the shape and sharpness of the indenter tip directly influence the size of the deformed region under the tip and also the gradient of the deformation, leading to changes in the strain localization.

Since the nanoindentation is in most cases performed using three-sided pyramid-shaped tips, the spherical or conical nanoindentation will not be explored in this work. The most used pyramidal tips are then Berkovich and cube-corner tips. The cube-corner tip, being the sharper one, leads to deeper indents with the same applied force. This, in combination with the fact that shallow indents are necessary for thin film nanoindentation, renders the cube-corner tip less useful for thin film nanoindentation. Therefore, the best and most used tip shape is the Berkovich pyramid. However, that does not rule out the possible exploitation of the tip shape. For very shallow indents, even a small change in the tip sharpness will have impact on the strain localization^50,51^, therefore, the tip sharpness and blunting process (due to the diamond wear^52^) should be tied to changes in thin film nanoindentation.

In this work, we compare the experimental nanoindentation into known nano-Cu/CuZr multilayer with three different tips. These tips differ only in the tip sharpness (tip radius), however, all the experiments have a proper tip calibration for the respective tip. These results are afterwards combined with a finite element (FE) simulation of the specific tips, showing the changes in the local strain fields and how the measured value is affected.

## Results

The results are divided into three sections. The first results are related to the investigation of the tips’ sharpness/bluntness and the calculation of the equivalent conical tip geometries, the second set of results are related to the experimental results from the nanoindentation experiments and the third section describes the numerical results.

### Tip shapes and dimensions

All of the tips were precisely calibrated in the nanoindenter via 100 indents into the fused silica. Fitting of the indents resulted in calibration function according to Eq. (1) in the Sect. 4.2 with the coefficients summarized in (Table 1). The tips are referenced according to their tip sharpness and named “Blunt” for the higher tip radius, “Standard” for a normal, usable tip radius and “Sharp” for a brand-new tip. For more information, see the Sect. 4.

**Table 1:** Coefficients of the calibration function (eq. (1)) for each tip.

	C_0_	C_1_	C_2_	C_3_
Blunt tip	11.2431	5101	−4.196·10^4^	6.747·10^4^
Standard tip	19.1889	1822	−3226	–
Sharp tip	20.9798	737	–	–

As the resulting calibration function coefficients in Table 1 show, there is a substantial difference between the three used tips. While the Sharp tip can be described by only twoparameter calibration function with the parameter *C*_0_ very close to the theoretical value for a tip with zero tip radius (with the theoretical value of 24.5 according to Doerner and Nix^53^). In contrast, the Blunt tip calibration function needs four coefficients for the correct tip calibration with the *C*_0_ value deviating extremely, confirming the large difference between this tip and ideal Berkovich geometry. The Standard tip calibration exhibits values of threeparameter calibration function as expected for a tip in use. The calibration functions according to Eq. (1) and parameters from Table 1 were used to recalculate the radius of the equivalent conical tip as a function of the contact depth *h*_c_ according to Eq. (3) in Sect. 4.3.

**Fig. 1:**
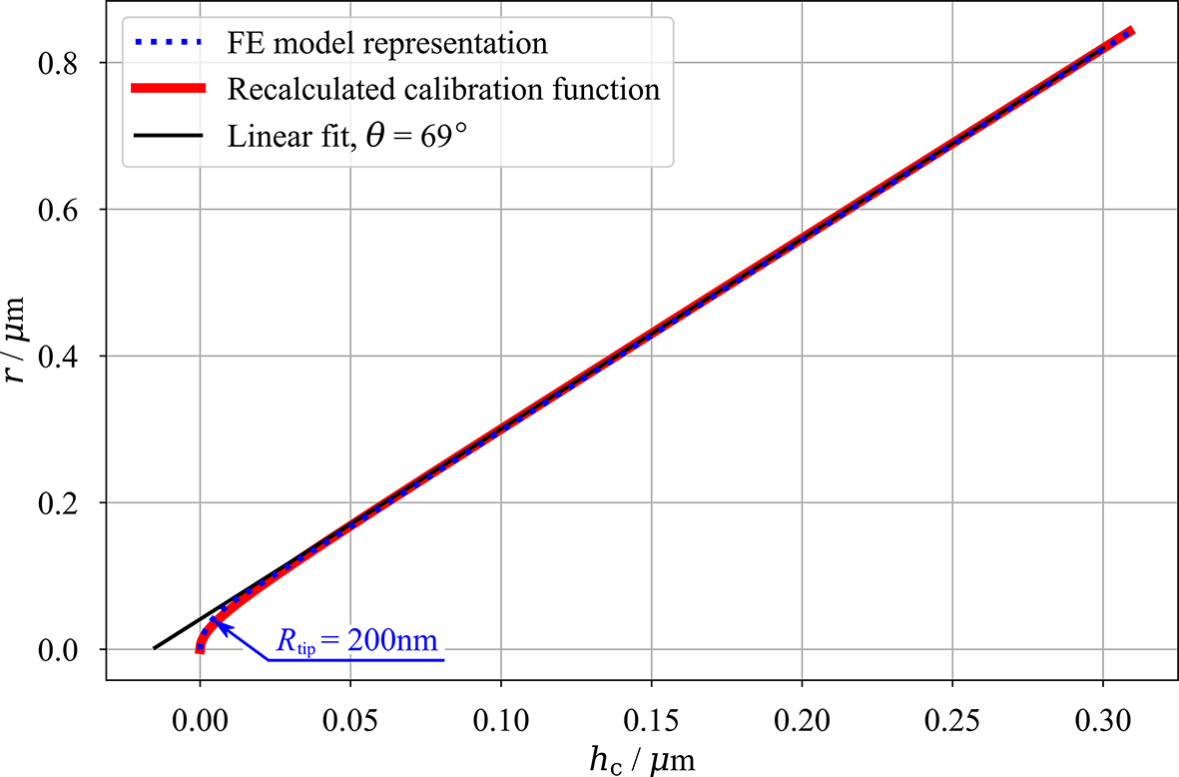
Example of the calibration function recalculation for the Sharp tip and fitting with linear function and tip radius *R*_tip_ (together with contour of the FE model tip profile), for the description of calibration function recalculation, see Sect. 4.3, Eq. (3).

The calibration function of the Sharp tip recalculated to the *r*(*h*_c_) function is presented as an example in Fig. 1 and the resulting values are: angles *θ* is 67º for the Blunt tip and 69º for both Standard and Sharp tips, the tip radii *R*_tip_ are 650 nm, 400 nm and 200 nm for Blunt, Standard and Sharp tip, respectively. It is obvious that all the tips deviate from the ideal equivalent conical tip angle. For the Sharp and Standard tip, the deviation is small and can be attributed to imperfections during the tip manufacturing process. However, the blunt tip equivalent angle of 67º deviates much more from the ideal value. This is a direct result of the tip wear and it shows that both tip radius and shape of the tip facets are affected. Since the calibration function was limited only to approximately *h*_c_ < 180 nm (while its validity can be extended beyond this limit, since the linear progression of the *r*(*h*_c_) is obvious even after this value, see Fig. 1), it cannot be postulated how far on the sides of the Blunt tip this effect extends. It should be noted that the Blunt tip calibration function did not show ideally linear progression for contact depth above 50 nm, therefore it is safe to assume that some light curvature is present on the faces of the Blunt tip. However, the investigation of actual tip shape is not the aim of this study, the linear fit for the equivalent conical tip was performed also for the Blunt tip and the fit with 67º angle was deemed the best, therefore, used in the FE simulation. These findings are well in line with the referenced research on the tip blunting^44^.

### Nanoindentation

The direct results obtained from the nanoindentation experiments were hardness *H* and reduced elastic modulus *E*_r_. As described in the Sect. 4.2, each tip was used to perform 100 individual indents into the nanoCu/Cu_60_Zr_40_ multilayer with a wide range of maximal load force. These results are plotted as a function of their contact depth *h*_c_ together with the values for the Si substrate and expected value of the multilayer (both taken from^45^).

**Fig. 2:**
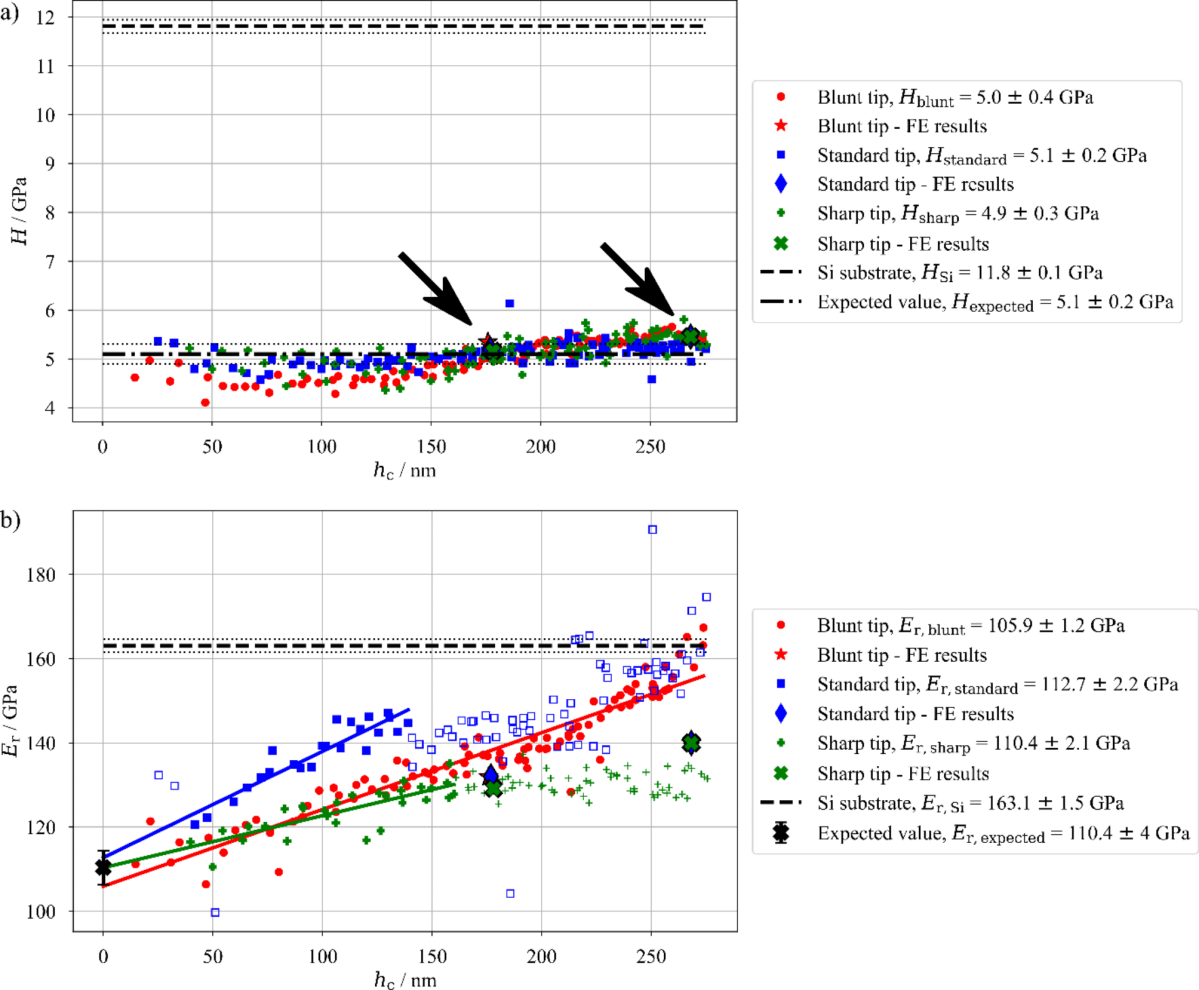
(**a**) Hardness *H* and (**b**) reduced elastic modulus *E*_r_ results for the nanoindentation with Blunt, Standard and Sharp tip as a function of the contact depth *h*_c_ (with reference values of *E*_r, Si_, *H*_Si_ and *H*_expected_, dashed and dashdotted lines, respectively, and their standard deviation marked with dotted lines as well as espected value of multilayer reduced elastic modulus *E*_r, expected_ from previous work^45^), results from FE models are for better visibility outlined in black color and in a) marked by black arrows.

As expected, the measured hardness values (shown in Fig. 2a) indicate no influence of the tip radius and there is no indication of the substrate effect for the hardness values whatsoever, confirming the previous findings about hardness (plasticity) being a short-range property (see^43,45^). Only the Blunt tip seems to result in lower hardness values in the shallow indentation regime for contact depths below 125 nm. Otherwise, all of the used tips show similar extent of the indentation size effect (contact depth below 50 nm) and the average values of hardness for each set at the same level as the expected value measured within the work in^45^. The hardness values obtained from the FE simulations coincide with the experimental data well, with no difference in regards to the modelled tip sharpness. More numerical results will be discussed in the next section.

The measured reduced elastic modulus *E*_r_ in Fig. 2b shows a large substrate effect, as expected. However, the results also show distinct differences between the three tips, despite the fact that all were properly calibrated and, in theory, should deliver the same results (for bulk material). All three result sets, when extrapolated by a linear function, lead to similar values of the multilayer reduced elastic modulus, with the Sharp tip showing the highest accuracy and Standard and Blunt tip over- and underestimating the results, respectively. All three tips seem to show smaller standard deviation of the fit than the expected value, since the results in^45^ were composed of four distinguished nanoindentation experiments, thus adding up to the deviation. However, for each tip a different portion of results has to be used for the extrapolation (full, bold markers in the graph). While the results for the blunt tip exhibit strong substrate effect with fully linear increase in measured *E*_r_ towards the Si value (pure Si elastic modulus is measured for indents with contact depth reaching 23% into the multilayer), both Standard and Sharp tips exhibit a non-linear progression. Both tips reach a plateau value, the Standard tip at the level of 143.4 GPa and the Sharp tip at the level of 130.4 GPa (being 63% and 38% in the gap between expected and substrate values, respectively). The measurements with the Standard tip start to increase linearly towards the Si value again at the contact depth of 220 nm, while the values measured with Sharp tip continue to stay on the same constant value (plateau) to the maximal possible contact depth. Values of *E*_r_ obtained from the FE simulations show similar progression, however, with much smaller differences between the respective tip radii. Numerical models with contact depth ~ 175 nm show both qualitatively and quantitatively comparable results with the experimental measurements, leading to roughly similar absolute values and, additionally, results for Sharp tip being the lowest, followed by the Blunt tip and Standard tip. However, when the contact depth reaches ~ 270 nm, the numerical results show increase in absolute values (in comparison to the lower *h*_c_), but almost no difference between different tips. This can be attributed to the general simplifications in the material model parameters in the FE approach (e.g. use of bilinear material model) and the tip shape simplification (axi-symmetrical model). These results are in good correlation with numerically obtained load-displacement curves, discussed in the next section. While the higher load model does not fully agree with the experimental measurements, it still shows similar trends in substrate effects.

### Numerical FE modelling

The numerical model allowed to quantify the stress and strain fields under the nanoindenter as well as record the differences between the load-displacement curves when only the tip shape changes (without any statistical error). Additionally, the comparison between experimentally obtained load-displacement curves and the numerical models could be visualized.

**Fig. 3:**
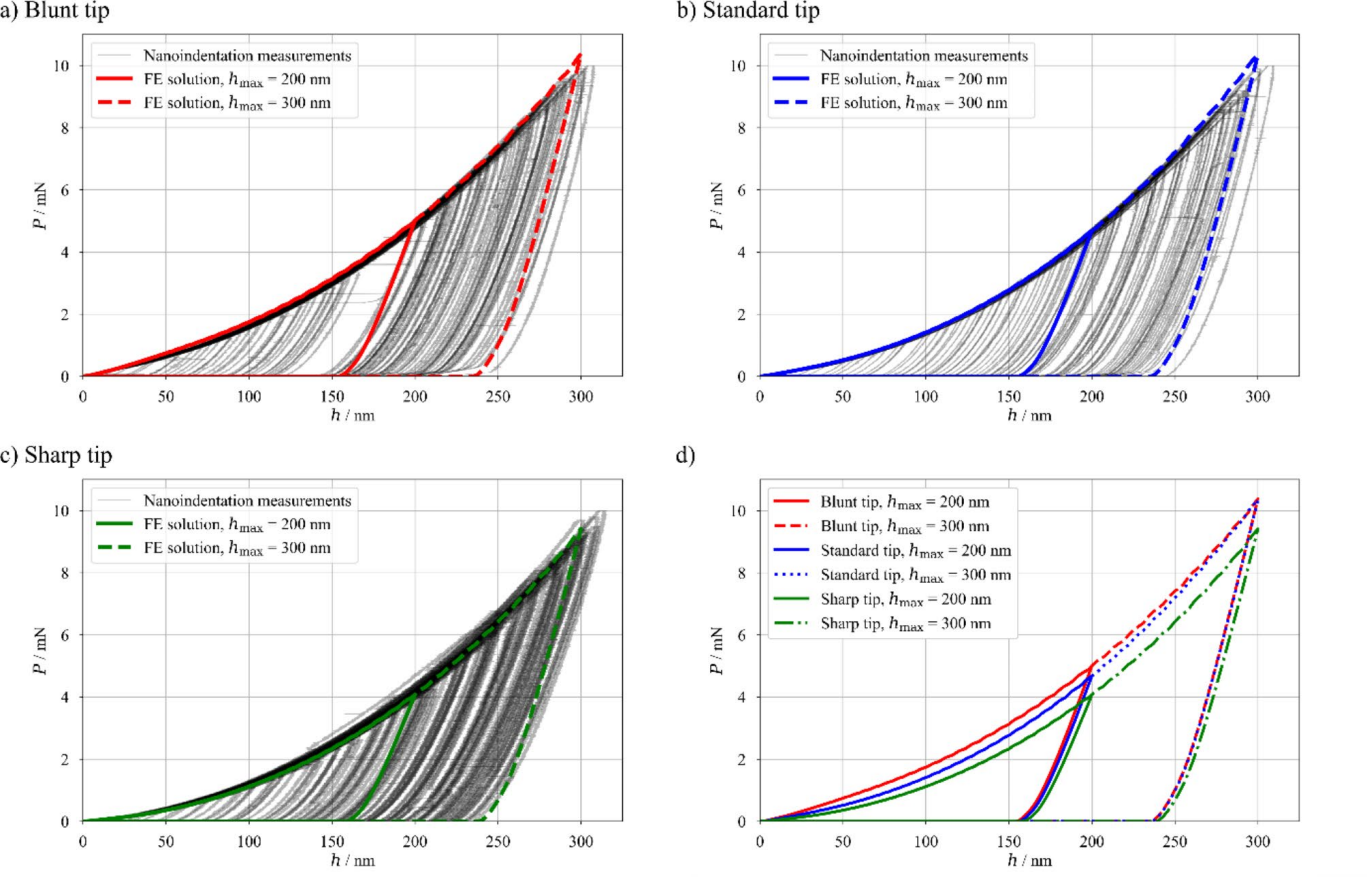
The comparison between experimentally obtained load-displacement curves and FE model results: (**a**) Blunt tip, (**b**) Standard tip, (**c**) Sharp tip and (**d**) all three tip shapes’ numerical results compared.

In Fig. 3, a good correlation between experiments and FE model is shown. The load-displacement curves from the numerical model match the experimental curves well. Therefore, the model can be deemed validated. A direct comparison between the three modelled cases (their load-displacement curves) is depicted in (Fig. 3d). The simulation with the geometry of the Sharp tip shows (in both maximal load cases) that the sharper tip needs less force to reach the same maximal depth as the blunter tips. That is also true for the comparison between Standard and Blunt tips for *h*_max_ = 200 nm. However, as the indentation goes deeper (*h*_max_ = 300 nm), the maximal force *P* needed to achieve the same indentation depth is almost identical for these two tips. Additionally, a strong difference in the curvature of the loading curve is clearly visible.

**Fig. 4:**
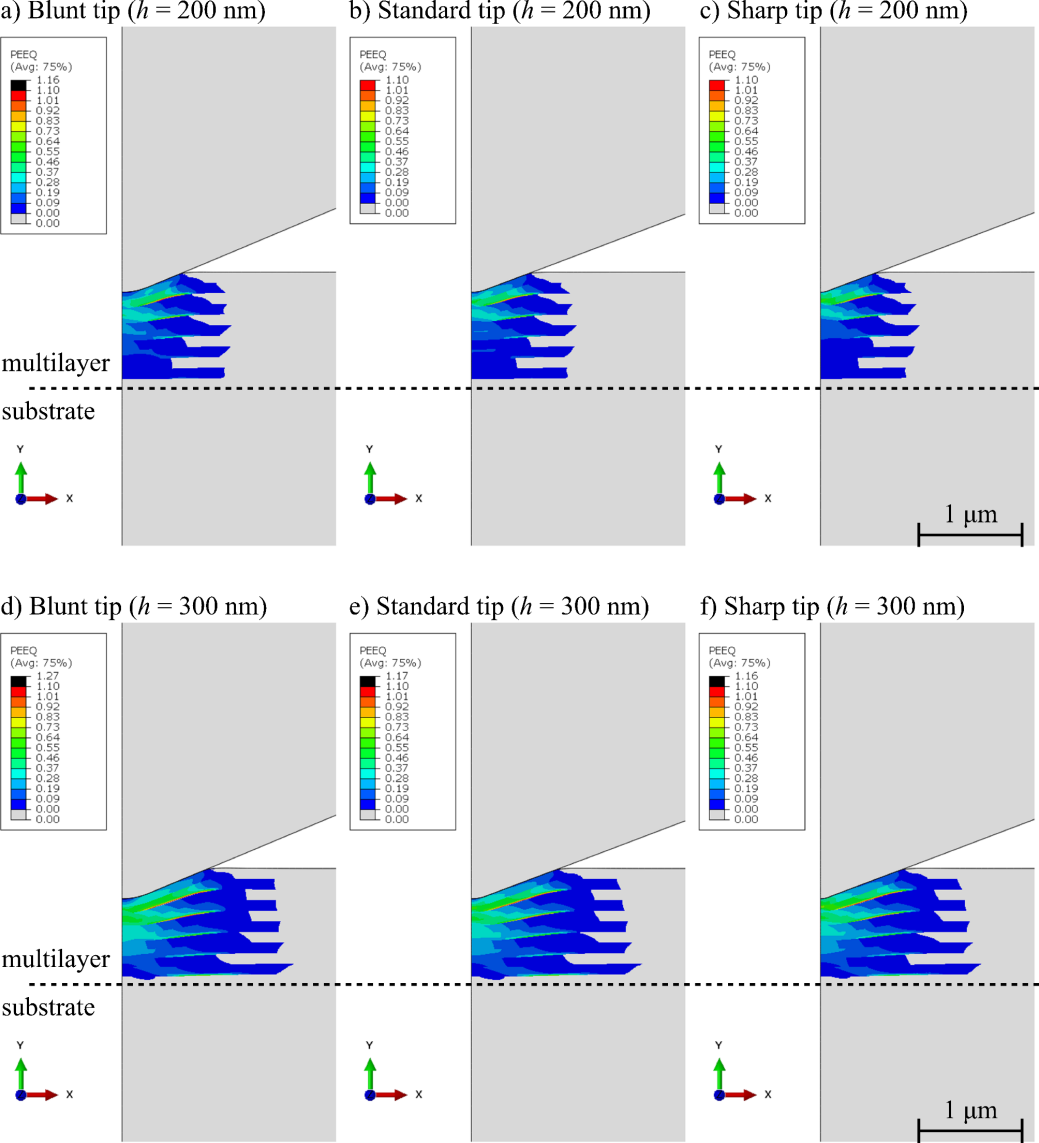
Equivalent plastic deformation (PEEQ) under the indenter tip from FE model, *h* = 200 nm: (**a**) Blunt tip, (**b**) Standard tip, (**c**) Sharp tip and *h* = 300 nm: (**d**) Blunt tip, (**e**) Standard tip, (**f**) Sharp tip.

The comparison of the equivalent plastic deformation field (PEEQ) under the indenter tip for an indentation depth of 300 nm for all three tips (see Fig. 4d–f) shows negligible differences in the extent of the plastic deformation under the tip. In all three cases, the plastic deformation field is well confined within the multilayers. Therefore, no substrate effect on hardness measurement is present. The bottom Cu_60_Zr_40_ layer is not even subjected to major yielding. The only visible difference between the tip geometries is the maximal plastic deformation, reaching higher values for the Blunt tip (*ε*_pl, Blunt_ = 1.27) than for Standard and Sharp tips (*ε*_pl, Standard_ = 1.17 and *ε*_pl, Sharp_ = 1.16, respectively). Note that the region of highest plastic deformation is not directly under the indenter tip, but shifted to the right side of each figure.

**Fig. 5:**
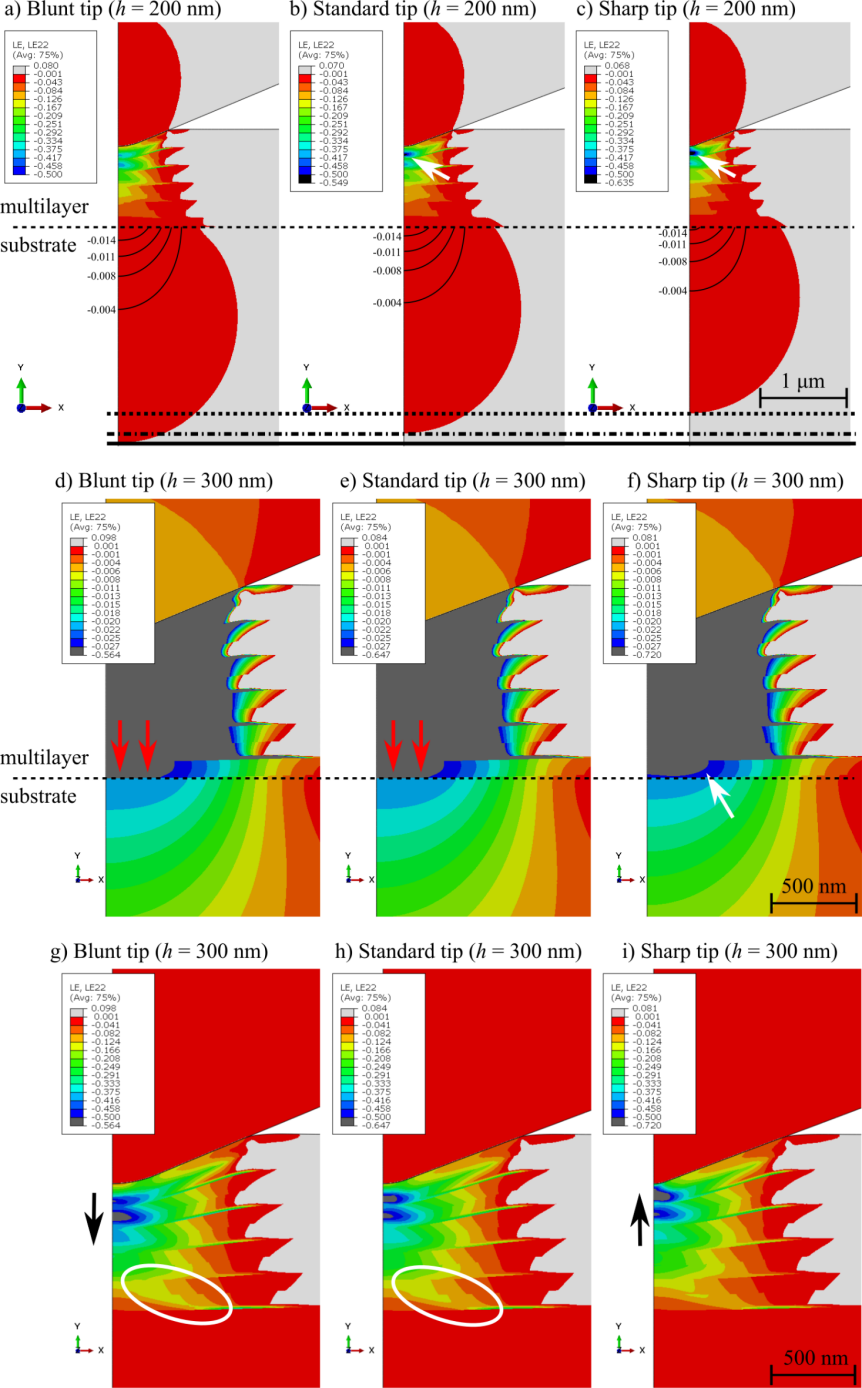
True (logarithmic) strain component in the y-direction (LE22) under the indenter tip from FE model, different maximal loading and color scale is presented: (**a**–**c**) *h* = 200 nm, besides the color-scale, additional contours are depicted in the substrate to improve the strain resolution, point of localization of the elastic deformation is marked by white arrows, (**d**–**f**) *h* = 300 nm, the colorscale is modified to show the extent of high compresive elastic strain through the botom Cu_60_Zr_40_ layer, red arrows mark the strain penetration to substrate interface and the white arrow marks the strain confinement in the multilayer, (**g**–**i**) *h* = 300 nm, the colorscale is same as in a–c), black arrows denotes the positional movement of the strain localization area in comparison to Standard tip case and white elipses show the strain field reaching towards the last Cu_60_Zr_40_ layer.

While the plastic deformation remained well-contained within the multilayer even for 300 nm deep indents, the elastic deformation field reached into the substrate even in the simulation with more shallow indentation depth of 200 nm (see Fig. 5). The true elastic compressive strain of 0.1% (the red contour in Fig. 5a–c) reaches more than two times deeper than the multilayer thickness. Moreover, the depicted strain reaches 1.5% in the substrate. Therefore, as expected, even shallow indents will show the substrate effect. The comparison between the different tip geometries also shows significant changes. The maximum extent of the 0.1% strain contour in Fig. 5a reaches the deepest position for the Blunt tip (marked by black full line), followed by a bit shallower extent with Standard tip (marked by black dash-dotted line) and the smallest elastic zone created by the Sharp tip (marked by black dotted line). On the other hand, in the multilayer, the sharp tip creates the highest gradient and localization of the elastic strain directly under the tip (at maximum of 6.8%), followed by the Standard tip with slightly higher value, but over much smaller area (at maximum of 7%), therefore the effective strain localization is smaller. For the Blunt tip, the strain field in the multilayer is much more uniform.

**Fig. 6:**
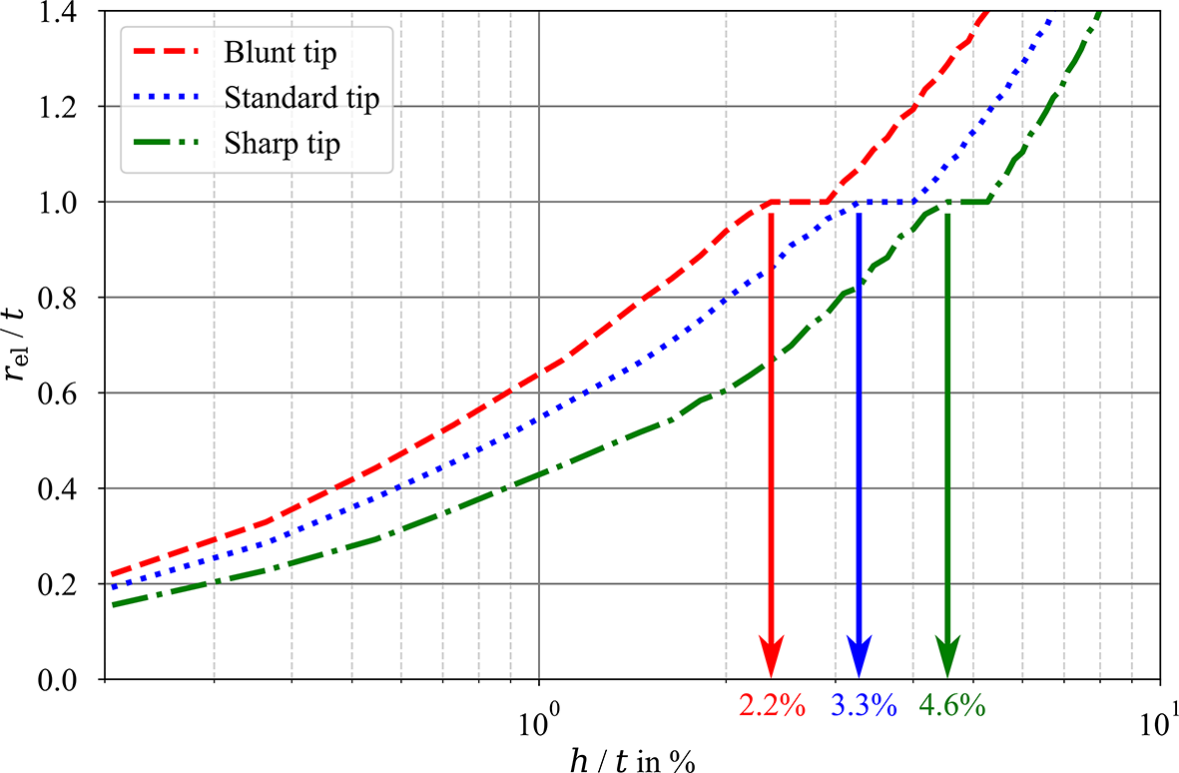
Dependence of the elastic strain field size on the indentation depth (FE models).

The growth of the elastic strain field size with the increasing indentation depth is depicted for all three tip geometries in (Fig. 6). The size of the elastically deformed region is described by the elastic zone radius *r*_el_ (the size of the elastic zone in the direction of nanoindentation, y-direction whereas the border of the elastic zone is considered to be the contour with 0.1% of compressive strain), normalized by the multilayer thickness *t*. Therefore, if *r*_el_ / *t* < 1, the elastic zone is within the multilayer, if *r*_el_ / *t* = 1, the elastic zone reaches the multilayer-substrate interface and if *r*_el_ / *t* > 1, the elastic zone reaches into the substrate and the nanoindentation measurement will be affected by the substrate. The indentation depth when the elastic zone reaches the substrate is different for the three tip geometries. As is also clear from the previous depiction of the elastic zones in Fig. 5, the elastic zone under the Blunt tip reaches the substrate first, when the indentation depth equals to 2.2% of the multilayer thickness. The elastic field under the Standard tip reaches the substrate for indentation depth of 3.3% of the multilayer thickness and with the Sharp tip, the indentation depth can reach up-to 4.6% of the multilayer thickness before it reaches the substrate.

## Discussion

As the results (both numerical simulations and nanoindentation experiments) show, the substrate effect is clearly present in the investigated material system and the results are in line with the previous works^32,34–41,43,45^.

The substrate did not affect the hardness measurement for the presented material system and in the range of the used contact depths. Moreover, there was no evidence of the changes in the hardness measurement when different tips were used (see Fig. 2a) and the actual mean values and standard deviations of the hardness measurement with each tip are comparable to the expected value from^45^. The only non-negligible difference between the different tips is the higher maximal value of the plastic deformation observed in the FE simulations for the Blunt tip (Fig. 4d). While the value of *ε*_pl_ for Blunt tip is higher by 0.1 than the *ε*_pl_ for the Standard tip, the overall shape of the plastic zone does not differ. The most likely reason for this difference is the fact that the Blunt tip has slightly different tip angle *θ*, meaning that the difference in the force needed for reaching the desired indentation depth is not only increased by the tip sharpness, but also the tip angle. Moreover, the difference in the tip angle changes the projected contact area for the designated indentation depth and, while the final hardness value is corrected by the proper tip calibration, the strain magnitude under the tip is different. Additionally, as is visible in Fig. 4d, the area with highest plastic deformation is always located not directly under the indenter tip, but slightly shifted to the side, indicating that it is not connected only to the tip radius, but also to the interaction between the sample and the tip sides due to their angle. There is only a slight increase in the hardness measurement for contact depths between 75 nm and 175 nm. This slight increase can be attributed to the multi-layered nature of the material system and is in a good agreement with the results from previous work^45^ – the plastic zone develops slowly under the indenter tip and grows through the layers one-by-one, including more and more interfaces. The presence of boundaries slightly increases the interfacial toughening for deeper contact depths, hence, the increase in the hardness. The effect of interfaces on the plastic zone size is supported also by the numerical simulations, showing that for indentation depths of 200 nm (see Fig. 4a-c) and 300 nm (see Fig. 4d-f), the plastic zone is already extended over all the interfaces (except the interface with the Si substrate), therefore, in such indentation depths, a plateau of hardness measurement is expected (as is visible in Fig. 2a for *h*_c_ larger than 175 nm).

The reduced elastic modulus measurements are, as expected, in a very good agreement with the previous works^43,45^ as well. In general, all three tips show a strong substrate effect for the *E*_r_ measurements, with its onset al.ready at shallow indents (*h*_c_ < 50 nm). This is supported by the numerical simulations, showing the large extent of the elastic deformation field, beyond the multilayer-substrate interface (see Fig. 5a-c) and the rapid growth of the elastic zone depicted in Fig. 6 where the elastic zone (0.1% elastic strain) reaches the substrate already for indentation depths between 2% and 5% of the multilayer thickness (indentation depths between 22 nm and 55 nm). However, the confirmation of the substrate effect on the reduced elastic modulus measurements is not the main focus of this work. The main focus is the influence of the Berkovich tip sharpness.

The nanoindentation results in Fig. 2b show that the tips, even when all were properly calibrated, lead to different outcomes. Therefore, the tip radius has a direct influence on the size of the deformation field under the indenter tip. Since the hardness measurements did not differ (when comparing the individual tips), but the reduced elastic modulus values are differing, it is safe to say that the main influence of the tip radius is directed towards the elastic deformation zone. In the Fig. 2b, the Blunt tip exhibits the most straightforward behavior. The measured *E*_r_ value with the Blunt tip linearly increases from the expected *E*_r_ value towards the value of Si and it reaches it, coincidently, at the maximum load available. Additionally, when performing the linear extrapolation towards *h*_c_ = 0 nm, the Blunt tip strongly underestimates effective reduced elastic modulus of the multilayer (*E*_r, blunt_ = 105.9 ± 1.2 GPa). Although the standard deviation of the fit is small, the underestimation of the result would not be acceptable. It can be explained by the steep angle of the linear fit and the fact that possibly the progression of the best fit function for this tip is not linear at the low contact depths. However, with almost negligible chance of getting precise results for such shallow indents, it is not possible to identify the ideal fit shape. In contrast to the Blunt tip, the Standard and Sharp tips exhibit a strongly non-linear progression of *E*_r_ vs. *h*_c_ results in Fig. 2b. The linear increase of the measured values from the true multilayer elastic modulus to the *E*_r, Si_ is abruptly stopped at the contact depths of 140 nm for the Standard tip and 160 nm for the Sharp tip. For the increase of the indentation depth, the results seem to stabilize at a plateau value (143.4 ± 3.0 GPa for the Standard tip and 130.4 ± 2.6 GPa for the Sharp tip). The Sharp tip results keep the plateau value until the maximal achieved indentation depth; however, the Standard tip results start again to linearly increase towards the Si value at a contact depth of approximately 220 nm. The onset of the plateau regime is clearly not connected directly to the elastic deformation field reaching the substrate. The elastic deformation reaches the substrate at indentation depths of 36 nm and 55 nm for the Standard and Sharp tip, respectively, therefore, much sooner than the start of the plateau regime. The plateau regime has to be connected with complex behavior of the growing elastic zone and not the pure fact of the deformation reaching the substrate. In comparison to other approaches, the observed experimental results are well in line with other authors’ observations. While the general transition between film and substrate values is expected, works by Tsui and Pharr^34^, Saha and Nix^35,36^, Saha et al.^37^ and Zhao et al.^41^ expect at least partially linear transition from the real thin film’s value to the one of the substrate, mostly for very small indentation depths. Moreover, works led by Saha^35–37^ lead to observations of formations of plateau values as well as the results in the presented work.

The FE simulation results point out several proofs, offering an explanation. First of all, the size and growth rate of the elastically deformed region (see Fig. 5a-c for the size and Fig. 6 for the growth rate) is a direct function of the tip radius. The Blunt tip with its radius of 650 nm exhibits the largest deformation zone and no plateau on the experimental results. This is followed by the Standard tip with its 400 nm of the tip radius and small plateau value between the expected and Si values and then with the Sharp tip and its 200 nm tip radius and seemingly infinite plateau in the measured results with value closer to the multilayer material property. The second observation is that the elastic deformation propagation is slightly hindered at the multilayer-substrate interface due to the difference in the true Young’s modulus (109.9 GPa for the Cu_60_Zr_40_ vs. 174.1 GPa for Si, see Table 2 in the Sect. 4.3). Due to the substrate having a larger elastic modulus, with the same applied force it exhibits a smaller value of the deformation. This is observable on the FE simulation results for indentation to the depth of 200 nm (Fig. 5a-c) and even better for *h* = 300 nm (Fig. 5d-f with detailed scale), where the extreme elastic deformation magnitude within the multilayer (4% and larger for compressive strain component in the direction of loading) is stopped at the Cu_60_Zr_40_ layer closest to the substrate, which is constrained by the Si substrate itself. There one can also see that the zone with the extreme deformation magnitude is largest again for the Blunt tip. However, for the simulation with indentation depth of 300 nm, both Blunt and Standard tips show increase of elastic strain within the bottom layer and the substrate (see Fig. 5g, h with modified contours). The almost identical shape of the elastic deformation field for the Blunt and Standard tips with indentation depth of 300 nm is also visible as the load-displacement curves for these tips almost merge for such indentation depth as shown in Fig. 3d.

Due to the above-mentioned hinderance of the elastic deformation and the localization of the deformation first in the multilayer and later on expanding into the substrate, the nanoindentation process of any thin layer, multilayer or coating on a stiffer substrate can be divided into four stages:

(I) Linear increase of the measured reduced elastic modulus.

- The elastic zone reaching the substrate and deformation density being comparable in both thin film and substrate.

(II) Plateau value of the measured reduced elastic modulus.

- The difference between the stiffnesses of the film and substrate hinders the deformation propagation in the substrate and the strain localization is constrained within the thin film.

(III) Further increase of measured reduced elastic modulus towards the substrate value.

- The strain localization cannot “fit” within the small deformation zone under the indenter and starts to rapidly deform also the substrate.

(IV) Pure substrate measurement.

- The deformation field and its localization exceed the thin film so the thin film is only bent into the substrate and the sample response measured by the nanoindenter equals to pure substrate measurement.

The onset and ending of each of the four stages of the thin film indentation is governed by the size and localization of the elastic deformation zone and these can be tuned via the different tip sharpness (or radii). The Blunt tip created during the indentation large elastically deformed region with a fairly uniform distribution both within the multilayer and the substrate (see Fig. 5a). Therefore, there is no separation and localization of the elastic strain maximum and the indentation experiment, thus the Blunt tip exhibits only the stage I) and IV) – a soft transition between the multilayer and substrate values of *E*_r_. The Standard tip, on the other hand, shows a small localization of the strain right under the tip, which is well-contained within the multilayer for the indentation depth of 200 nm (see Fig. 5b). Therefore, the initial stage of uniform deformation and observation according to the stage I) is followed by the plateau stage II). The deformation localization throughout the further loading starts to also influence the substrate, leading to the observation of the stage III). Since for the Standard tip the maximal used indentation depth showed results comparable to *E*_r, Si_, it is safe to assume that further, deeper indentation would lead to full substrate measurements. Lastly, the Sharp tip exhibits only stages (I) and (II) in the Fig. 2b. This is caused by a strong strain localization under the indenter for both indentation depths of 200 nm (see Fig. 5c) and 300 nm with negligible interaction with the substrate (see Fig. 5f, i). It has to be noted that the actual parameters’ combination and quantification of how they influence the elastic zone growth under the tip cannot be extracted from the presented setup with only one sample type. More thorough research will be performed in future. However, the comparison of three vastly different tips shows the trends in the indentation which were not observed before and points out the importance of knowing the tip sharpness.

The actual influence of the tip radius is, as described above, connected through the elastic deformation to the slope of the change of the apparent reduced elastic modulus (from true thin film value to the substrate) as well as to the appearance of the plateau stage II). This can be easily exploited for more precise and less time-demanding indentation experiments. In contrast to the previous work on the same material system where approx. 400 indents were used to obtain the effective multilayer elastic modulus, in this work, only 100 indents (and only half of the points used for the linear fit) with the Sharp tip were necessary to obtain the same value of the elastic modulus with considerably smaller standard deviation. This implies that, even if the tip radius might not be necessary to track for bulk materials nanoindentation (where large indentation depths are possible), when the measured volume of the sample needs to be small, a sharp tip has to be used for accurate results. The sharp tip is crucial for localization of the deformation in a small volume.

## Methods and materials

### Material system for nanoindentation

**Fig. 7:**
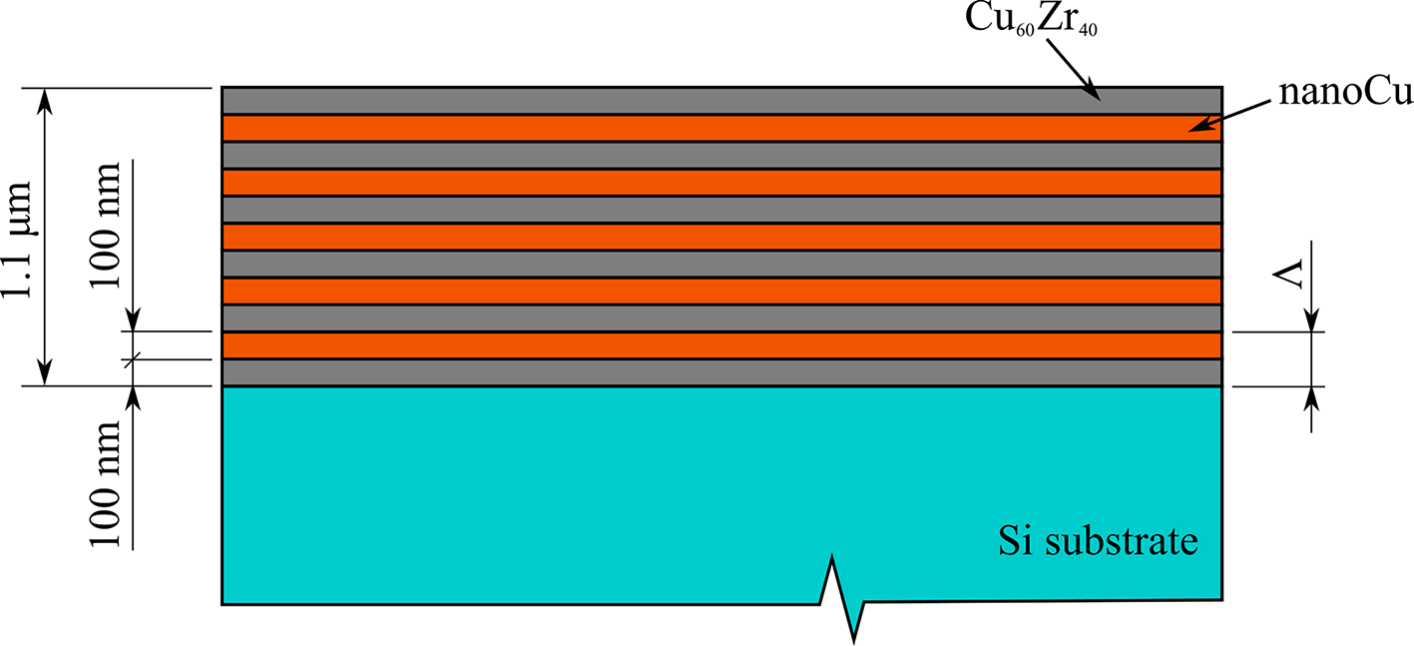
Schematic of the nanoCu/Cu_60_Zr_40_ multilayer used in the experiments.

As a material system for testing of the influence of the tip sharpness, the multilayer consisting of nanocrystalline Cu (nanoCu) and Cu_60_Zr_40_, same as in a previous publication^45^, was used. This multi-layered system was suitable for this type of testing since their properties were already well known and measured in our laboratory. From the four different multi-layered sample types used in^45^ only one was used in this study – the nanoCu/Cu_60_Zr_40_ with the periodic bi-layer thickness of Λ = 200 nm containing 11 layers in total, forming a multilayer with overall thickness of 1.1 μm (with Cu_60_Zr_40_ being the starting layer closest to the Si substrate and capping the whole system at the free surface, depicted in Fig. 7).

The used sample was synthesized using direct current magnetron sputtering within a lab-scale modular PVD deposition chamber equipped with two sputtering sources (Korvus Technology) using two elemental targets with a diameter of 50.8 mm and a thickness of 3.2 mm (a 99.999% Cu target and a 99.95% Zr target, both provided by HMW Hauner). Before sputtering the base pressure of the deposition chamber was set to below 2.6 × 10^–5^ mbar and throughout all depositions the working gas (Ar) pressure was set to 1.7 × 10^–3^ mbar. The Si substrate was attached to a sample stage rotating with a frequency of 20 rounds/minute. The nanoCu layers were deposited at 40 W target power yielding sputtering rates of 10 nm/min. The Cu_60_Zr_40_ layers were created using co-deposition of both pure metal targets, where the Cu target power was set at 40 W and the Zr target power was set to 95 W, leading to a deposition rate of 20 nm/min. For more information on the sample microstructural analysis, the reader is referred to the original publication^45^, the detailed information is not crucial for the purposes of this manuscript. The described sputtering parameters for the Cu and CuZr layers yielded fine grained Cu and amorphous Cu_60_Zr_40_ layers, respectively.

### Nanoindentation procedure

The nanoindentation measurements were performed using a TS77 Select Bruker-Hysitron nanoindentation platform. To quantify the influence of the tip radius on the thin films nanoindentation, three different Berkovich tips were selected. The first tip (henceforth called “Blunt tip”) was a fairly used one (it was used as a regular tip for a long time and numerous indentations, approximately 48 000 individual indents into various metallic and non-metallic materials), at the end of its life expectancy. This tip should not be used for any new standard, precise nanoindentation, since its tip radius can be considered too large according to the nanoindentation ISO-14,577 standard^54^. However, for the purpose of this work, it can serve as an example of a blunt tip. As the second tip (henceforth called “Standard tip”), a standard, currently-in-use tip was selected. This tip is not new, it has undergone several indentations (approximately 4 700 individual indents into various metallic and non-metallic materials), however, it is not blunt and still in a fairly good shape. The third tip (henceforth called as “Sharp tip”) was picked as a brand-new, freshly unpacked from the original packaging from the manufacture. This tip has not seen any nanoindentation prior the experiments for this work (with the exception of the necessary 150 calibration indents), thus it is deemed to be as sharp as possible. The actual tip shape and tip radius for each tip was evaluated from the tip area calibration functions by matching it with equivalent conical shaped tip model and the resulting values are presented in the results section.

Prior each testing indentation, all three tips were calibrated within the machine. First, after each tip change, the frame compliance was calibrated using 25 open-loop indents into fused silica (with the loading ranging from 5 mN to 10 mN). Afterwards, the tip area calibration function was created using 100 open-loop indents into fused silica (with loading ranging from 25 µN to 10 mN). This resulted in calibration indents with contact depths between 5 nm and 180 nm, limiting the calibration function boundaries. The tip area calibration functions themselves were obtained through reverse Oliver-Pharr method^22^ with known elastic material properties of the tip (Young’s modulus *E*_tip_ = 1140 GPa and Poisson’s ratio *ν*_tip_ = 0.07) and fused silica (Young’s modulus *E*_FS_ = 72 GPa and Poisson’s ratio *ν*_FS_ = 0.17) by fitting the results with the calibration function:

1 $$\:{A}_{\text{c}}={C}_{0}\cdot\:{h}_{\text{c}}^{2}+{C}_{1}\cdot\:{h}_{\text{c}}+{C}_{2}\cdot\:{h}_{\text{c}}^{0.5}+{C}_{3}\cdot\:{h}_{\text{c}}^{0.25}$$

where *A*_c_ is the projected contact area, *C*_i_ (i = 1, 2, 3 or 4) are the calibration function coefficients and *h*_c_ is the contact depth.

With each of the three tips, a total of 100 force-controlled indents in a 10 × 10 pattern with spacing of 10 μm between the indents were made into the nanoCu/Cu_60_Zr_40_ multilayer. Each indent had different maximal load, ranging from 25 µN to 10 mN. This procedure provided enough of the datapoints within a reasonable range of the contact depths. The substrate indents were not performed, instead, the values from the previous publication^45^ were used, since the indented sample was the same as in the previous work. For analysis, standard Oliver-Pharr method^22^ was used in conjunction with the previously calibrated function from Eq. (1), for each corresponding tip. The actual elastic modulus *E* of the indented material can be then extracted from the resulting reduced elastic modulus *E*_r_ via well-known relation:

2 $$\:\frac{1}{{E}_{\text{r}}}=\frac{1-{\nu\:}_{\text{tip}}^{2}}{{E}_{\text{tip}}}+\frac{1-{\nu\:}^{2}}{E}$$

However, with unknown value of the Poisson’s ratio of the multilayer *ν* (and for the sake of simplicity), only the reduced elastic modulus *E*_r_ will be used for results analysis and Eq. (2) will not be used.

### Numerical finite element (FE) modelling

To evaluate the stress and strain evolution under the nanoindenter tip and to compare them (if any differences are present) for tips with different tip radii, the numerical FE model of the nanoindentation was created within the Dassault Systèmes Abaqus code (in version 2018).

Since the modelled case is a Berkovich tip, a 3D problem (with use of only two possible symmetry planes, similar to models used in^43^) has to be modelled. However, since Berkovich tip is a self-similar tip and can be modelled with equivalent conical tip^52^, for a faster simulation of the nanoindentation and more straightforward modelling of the tip radius, a 2D, axi-symmetrical model of an equivalent conical indenter tip was modelled.

**Fig. 8:**
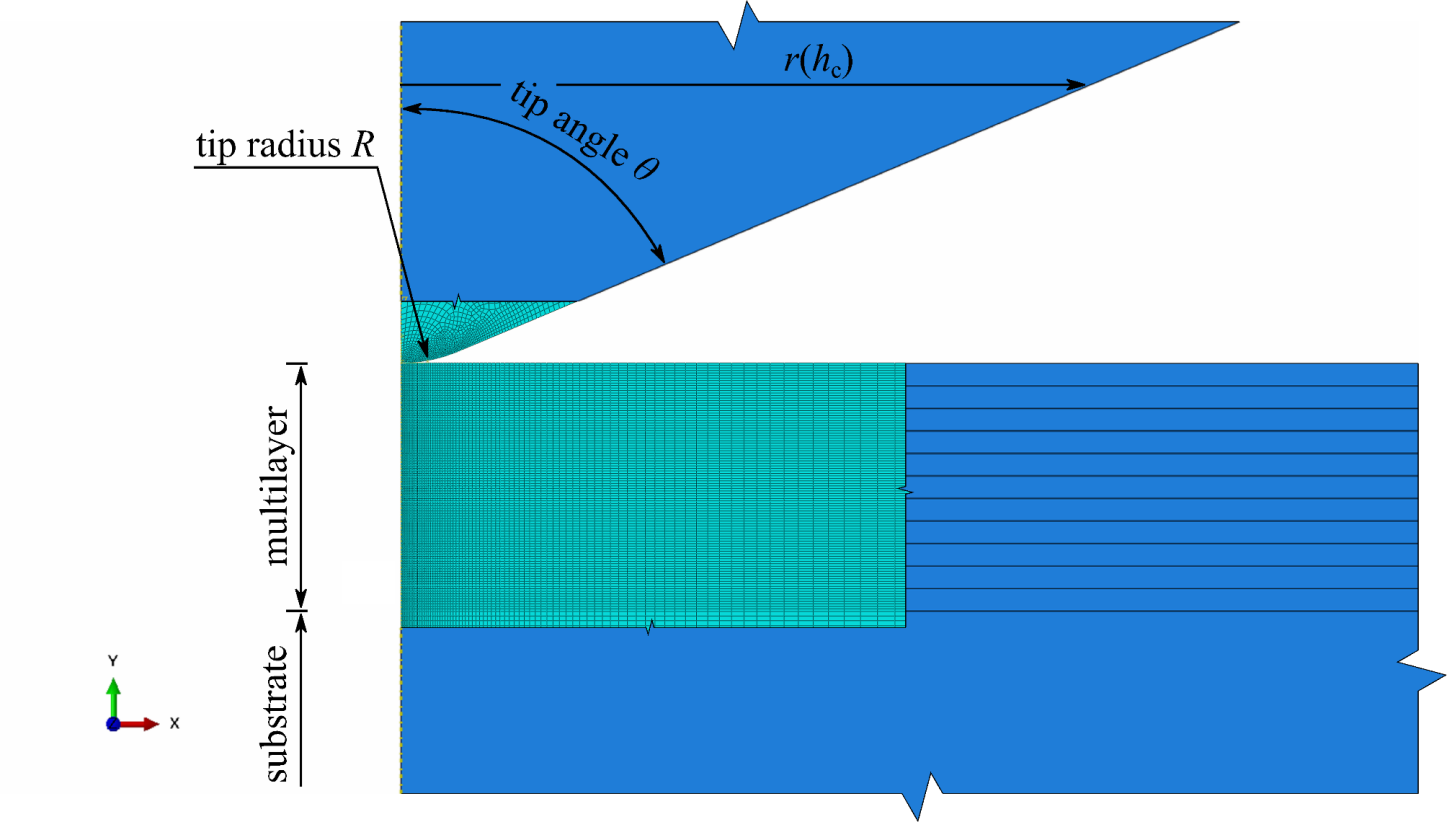
Geometry and the mesh of the FE model.

The geometry of the axi-symmetrical FE model with the conical tip, multi-layered section and necessary parameters described is shown in Fig. 8. The tip radius *R* was the main parameter to fit the three tip shapes. To have a proper equivalent tip geometry, in case of cone shape, the tip angle should be selected according to the actual 3D tip shape. A sharp Berkovich tip should be equivalent to a conical tip with the angle *θ* = 70.1º^52,54^. However, the study on the nanoindenter tip wear showed that not only the tip radius changes during the tip lifetime, but also the angles of the Berkovich tip faces can be slightly different, meaning also a different equivalent angle *θ* on the conical tip. Since especially the worn tip could be subjected to tip angle alterations, the angle *θ* was taken as the second parameter adjusted according to the real tip geometry when constructing the FE model. The calibration of the *R* and *θ* parameters was performed through the known tip area calibration function (for each tip separately) using Eq. (1). To assure the model tip equivalency, the projected contact area of both real Berkovich tip and equivalent conical tip must be the same, therefore:

$$\:{A}_{\text{c}}={A}_{\text{c,\:{equivalent}}}$$

3 $$\:{C}_{0}\cdot\:{h}_{\text{c}}^{2}+{C}_{1}\cdot\:{h}_{\text{c}}+{C}_{2}\cdot\:{h}_{\text{c}}^{0.5}+{C}_{3}\cdot\:{h}_{\text{c}}^{0.25}=\pi\:\cdot\:{r}^{2}$$

$$\:r\left({h}_{\text{c}}\right)=\sqrt{\frac{{C}_{0}\cdot\:{h}_{\text{c}}^{2}+{C}_{1}\cdot\:{h}_{\text{c}}+{C}_{2}\cdot\:{h}_{\text{c}}^{0.5}+{C}_{3}\cdot\:{h}_{\text{c}}^{0.25}}{\pi\:}}$$

With the equivalency of the tip contact areas *A*_c_ and *A*_c, equivalent_, the actual size of the cone (in terms of its radius *r*(*h*_c_) as a function of contact depth *h*_c_) could be calculated according to Eq. (3). Since the used Berkovich tips are not ideal and they contain manufacturing imperfections as well as damage from the tip use, the function *r*(*h*_c_) is not an ideal cross-section of a half-cone, but it could be easily fitted with a linear function (for the side of the cone) and a circle at the tip (for the rounded tip). The actual calculated values are presented in the results section.

In Fig. 8 the detail on the FE mesh of the multilayer and the tip is visible. The model was meshed with CAX4–4-node bilinear axisymmetric quadrilateral elements^55^. Each layer from the multilayer consisted of 9 elements through its thickness (element size of 11.1 nm) and the element width linearly increasing from 5 nm at the indented area (axis of symmetry) to 2.5 μm at the far end of the model. The tip was meshed with the equal size of elements as the top of the sample to keep the contact between similarly sized elements. Substrate was meshed in the same manner as the layers in x-direction and in the y-direction, the element size was set to linearly increase from 20 nm at the interface with Cu_60_Zr_40_ to 5 μm at the bottom of the model (substrate thickness was set to 98.9 μm which is thick enough to not have an influence of the bottom side of the substrate on the results).

For the material models, the nanoindenter tip was assumed to be purely elastic (described only by its elastic modulus *E* and Poisson’s ratio *ν*). All other materials involved were modelled with a bi-linear, elastic – ideally plastic material model (described by their elastic modulus *E*, Poisson’s ratio *ν* and yield stress *σ*_y_). The yield stresses of the respective materials were calculated using well-known Tabor’s relation (*σ*_y_ ≈ *H*/3, the ratio can vary depending on material, see^56^) from hardness values measured in a similar fashion as in^45^. Used parameters are presented in Table 2.

**Table 2:** Material parameters of the FE model.

Material	*E* / GPa	*ν*	*σ*_y_ / GPa
tip (diamond)	1140	0.07	–
substrate (Si)	174.1	0.22	4.15
nanoCu	112.2	0.34	1.20
Cu_60_Zr_40_	109.9	0.34	2.70

The FE model inherently included the axi-symmetric boundary condition with the axis of symmetry being the y-axis. Additionally, the bottom edge of the substrate was set to have zero displacement in the y-direction (far enough in the distance of 100 μm from the top of the sample) and a coupled boundary condition on the right side of the model to create a symmetrylike boundary condition, modelling the semi-infinite case. The loading was managed through the prescribed displacement of the top edge of the indenter tip by 200 nm and 300 nm (for two versions of the simulation with two maximal loads) in the negative y-direction followed by unloading back to starting position. The contact between the sample and tip was modelled as a surface-to-surface frictional contact with “penalty” contact definition and coefficient of friction set to 0.15, which is a standard value used for diamond-metal contacts^57–60^.

## Conclusions

The presented work showed that, while still present, the substrate effect in thin film nanoindentation can be reduced/modified with use of sharper indenter tip (smaller tip radius). Sharp tips can produce the elastic strain localization and force the majority of deformation to happen inside the measured thin film, thus making the actual experimental procedure easier for the dissemination of results. The experiments and numerical simulation tied the strain localization to the experimental results and, moreover, showed the existence of the plateau of measured elastic modulus as a function of contact depth for certain conditions, whereas sharper tips promote the existence of such plateau. Further research on the actual conditions minimizing the strain localization area will be subject to future research.

## Data Availability

The raw data and code from analysis will be provided by author (S. Zak, stanislav.zak@oeaw.ac.at) upon a reasonable request.
